# Association of R1939W and P1987R variants of *Otoferlin* (*OTOF*) gene with severe to profound nonsyndromic sensorineural hearing loss in Pakistani subjects

**DOI:** 10.12669/pjms.39.5.6393

**Published:** 2023

**Authors:** Hammael Naseer, Amir Rashid, Asifa Majeed, Zunaira Ali Baig

**Affiliations:** 1 Dr. Hammael Naseer, MBBS. Department of Biochemistry and Molecular Biology, Army Medical College, National University of Medical Sciences, Rawalpindi, Pakistan; 2 Dr. Amir Rashid, PhD. Department of Biochemistry and Molecular Biology, Army Medical College, National University of Medical Sciences, Rawalpindi, Pakistan; 3 Dr. Asifa Majeed, Post Doc. Department of Biochemistry and Molecular Biology, Army Medical College, National University of Medical Sciences, Rawalpindi, Pakistan; 4 Zunaira Ali Baig, M.Phil. Department of Biochemistry and Molecular Biology, Army Medical College, National University of Medical Sciences, Rawalpindi, Pakistan

**Keywords:** Nonsyndromic deafness, Sensorineural hearing loss, *Otoferlin* gene, R1939W variant, P1987R variant, Consanguinity, Cochlear implant

## Abstract

**Objective:**

To find possible association of R1939W and P1987R variants of *OTOF* gene with severe to profound NSSHL in cochlear implant subjects.

**Methods:**

It was a case control study, conducted from June 2021 to February 2022, comprising 50 cases of severe to profound NSSHL who had received cochlear implant from ENT Department, CMH Rawalpindi and 50 age-matched healthy controls from PEMH Rawalpindi. Blood samples were collected from all the subjects, followed by DNA extraction and allele-specific polymerase chain reaction, performed at Multi-disciplinary Laboratory of Department of Biochemistry and Molecular Biology, Army Medical College Rawalpindi. Statistical analysis was done using ‘SPSS’ and ‘XLSTAT’, followed by genetic analysis using ‘SNPstat’.

**Results:**

Mean age of the cases was 5.96 ± 4.62 years (*N*=50), comprising 58% males and 42% females. All had bilateral and prelingual HL. Parental consanguinity was 72%, whereas 62% cases had a positive family history of deafness. Alleles of R1939W and P1987R were not associated with NSSHL, as shown by their *p* values of 0.56 and 0.89 respectively. For R1939W ORs were 0.71 (dominant model) and 0.80 (overdominant model), indicating negative association with NSSHL. Regarding P1987R OR was 0.96 (log-additive model). Genotypes of both variants were not in HW Equilibrium (*p* <0.0001), whereas their alleles showed high LD (*D’*=0.92).

**Conclusion:**

High percentage of parental consanguinity was observed among cochlear implant candidates. The *OTOF* variants R1939W and P1987R were found to have protective roles against NSSHL in study population.

## INTRODUCTION

Hearing loss (HL) is a globally prevalent, multifactorial, and polygenic condition. About 124 genes have been identified to be associated with nonsyndromic HL, and 78 of them are inherited in an autosomal recessive manner.^1^ Worldwide, 1.5 billion people suffer from hearing impairment, which by the year 2050 is estimated to reach 2.5 billion.^2^ According to an estimate, 736,900 cochlear implant surgeries have been performed around the globe, by the year 2019.^3^ In Pakistan, 244,254 individuals suffer from deafness.^4^

Certain environmental^5^ and genetic factors play their roles in the onset of the disease. Consanguinity increases the susceptibility of HL, owing to reduction in genetic diversity. Pakistan has a high prevalence of consanguineous marriages (70%).^6^ Various genetic studies have been carried out to explore their associations with HL. *Otoferlin* (*OTOF*) gene located on chromosome 2p23.3 (48 exons), is associated with nonsyndromic autosomal recessive deafness, DFNB9.^7^ It is mainly involved in the fusion of synaptic vesicles with plasma membrane of the inner hair cells of cochlea and release of neurotransmitter at the ribbon synapse,^8^ participating in conduction of sound signals to the brain.

Numerous studies have investigated the role of *otoferlin* gene in hearing impairment. A Chinese study determined 25 pathogenic variants of *OTOF* gene including p.R1939W in 34 subjects suffering from Auditory Neuropathy Spectrum Disorder.^9^ A genetic study involving Muslim families from Indian Jammu and Kashmir sequenced *OTOF* gene and found c.2122C>T mutation to be prevalent among the cases.^10^ In Pakistan, polymorphisms of about 57 deafness associated genes have been identified, where 47% of moderate to severe HL is contributed by six genes i.e., *OTOF, GJB2, TMC1, MYO15A, SLC26A4*, and *TMPRSS3*.^11^ Mutation analysis of 21 Pakistani Punjabi subjects suffering from nonsyndromic HL, with history of consanguinity, revealed two novel pathogenic variants; c.4990-4991del and c.2443delC in *OTOF* gene.^12^ Another study conducted on a Pakistani family comprising seven subjects with autosomal recessive HL found pathogenic *OTOF* variant c.3289-1 G>T as a risk factor for the disease.^13^ In view of these significant associations between *OTOF* gene and hereditary deafness, it is critical to provide evidence-based information on the prevalence of *OTOF* variants in Pakistan and their association with the disease. So that a national genetic database on hereditary deafness could be established and extensive genetic testing be provided.

## METHODS

The study was conducted at Multi-Disciplinary Laboratory of Department of Biochemistry and Molecular Biology, Army Medical College Rawalpindi, in collaboration with ENT Department, Combined Military Hospital (CMH) Rawalpindi and Chemical Pathology Laboratory, Pak Emirates Military Hospital (PEMH) Rawalpindi. It was a case control study, from June 2021 to February 2022.

### Ethical Approval:

A formal approval from Ethical Review Committee of Army Medical College was taken before commencement of the study (ID#148, dated 15^th^ June 2021).

Total sample size was 100, calculated by WHO sample size calculator with deafness prevalence of 0.12%^4^ (95% *CI* and 5% error). Participants were selected using nonprobability purposive sampling technique. Case group comprised 50 individuals suffering from severe to profound nonsyndromic sensorineural hearing loss (NSSHL). All cases had received unilateral cochlear implant (CI) from CMH, Rawalpindi. Control group comprised 50 age-matched healthy individuals (both genders). Five ml venous blood samples were collected from all study participants, under aseptic measures, after taking informed written consent.

### Molecular Analysis:

Extraction of DNA was done by organic method^14^ followed by gel electrophoresis (1% agarose). *OTOF* gene information was obtained from Online Mendelian Inheritance in Man (https://www.omim.org/entry/603681). The sequence of exon 47 of the OTOF gene was downloaded from National Center for Biotechnology Information (https://www.ncbi.nlm.nih.gov/nuccore/224465243). Primers for the two variants were designed using online bioinformatics tool WASP (Web-based Allele Specific Primer), (https://bioinfo.biotec.or.th/WASP/home). The sequence of R1939W wildtype forward primer was 5’CTCTCTTCCACTTCCCAGAC3’, mutant forward primer was 5’CTCTCTTCCACTTCCCAGAT3’, and common reverse primer was 5’GATCTTGATGATGAGCCACT3’, with product size of 112bp. For P1987R, wildtype forward primer was 5’GCTCTTCCTCTACAGCCTCTC3’, mutant forward primer was 5’GCTCTTCCTCTACAGCCTCTG3’, and common reverse primer was 5’AGATGGGAAAGAGTCCAAG3’ with product size 129bp.

Allele-Specific Polymerase Chain Reaction (AS-PCR) was carried out to detect the two variants. The protocol comprised of two reactions: the first involved wildtype forward primer and the second involved mutant forward primer. Reverse primer was common in both reactions. The reaction mixture was prepared using Taq buffer (1X) 2.0μl, MgCl_2_ (2mMol) 1.6μl, dNTPs mix (0.2mMol) 0.4μl, forward primer (1pmol/μl) 0.8μl, reverse primer (1pmol/μl) 0.8μl, Taq DNA polymerase (1 unit) 1.0μl, DNA template (200ng) 1.0μl, nuclease free water 12.4μl. AS-PCR parameters for R1939W were: hot start at 95°C for five minutes, denaturation at 95°C for 30 seconds, annealing at 58.8°C for 30 seconds, extension at 72°C for 30 seconds, and final extension at 72°C for seven minutes. Similar protocol was used for P1987R except for annealing temperature that was set at 61.1°C. Total 35 cycles were run. At the end, gel electrophoresis (2% agarose) was performed with the PCR products to detect the wild/mutant alleles.

### Statistical Analysis:

Deafness associated parameters were analyzed using SPSS (version 22). Age was expressed as mean ± standard deviation. Categorical data was exhibited as frequency percentages. The two genetic variants were analyzed using online bioinformatics tool SNPstat (available at: https://www.snpstats.net/start.htm). In order to determine the association of *OTOF* gene variants with NSSHL, Pearson’s Chi square test and odds ratio (OR) were used. For the correlation analysis, Cochran-Armitage Trend test was applied using another statistical software XLSTAT (version 2022.5.1). To test for Hardy-Weinberg Equilibrium (HWE), Exact test was applied, and Linkage Disequilibrium (LD) was calculated for the two variants using haplotype frequency given by SNPstat. *P*-value < 0.05 was considered significant.

## RESULTS

All cases had severe to profound sensorineural HL, diagnosed by Auditory brainstem response (ABR) and brainstem evoked response audiometry (BERA). Physical examination, tympanometry, CT scan and MRI (brain) were unremarkable for any external/middle ear pathology or associated syndromic malformations. Those cases who had used hearing aids before cochlear implant reported no benefit. Cases visited ENT OPD for activation/mapping of implant device, and/or post-implant speech therapy.

**Fig.1:**
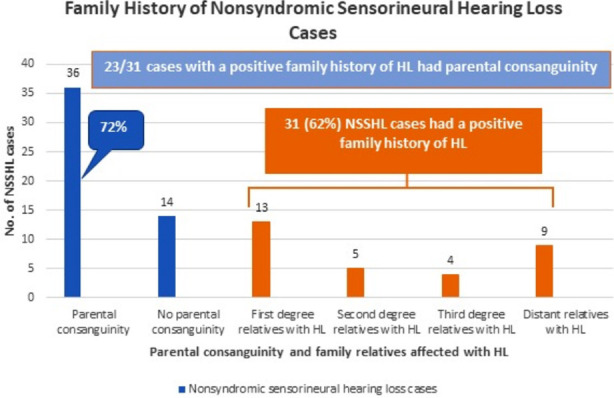
Family history of nonsyndromic sensorineural hearing loss (NSSHL) cases, (N=50).

Mean age of the cases was 5.96 ± 4.62 years. There were 29 (58%) males and 21 (42%) females. All cases had bilateral and prelingual onset of HL, 47 (94%) had non-progressive and 3 (6%) had progressive disease course. Milestones’ development was normal in 33 (66%) and delayed in 17 (34%) cases. Fig.1 shows family history of NSSHL cases.

Mean marital age of the cases’ mothers was 22.10 ± 3.14 years and that of the fathers was 26.65 ± 3.73 years. Most of the cases were of Punjabi ethnicity- 34 (72.34%), followed by Pathan-9 (19.15%), Balochi-2 (4.26%), Sindhi-1 (2.13%), and Siraiki-1 (2.13%). Regional distribution showed that 30 (61.22%) cases were from Punjab, 12 (24.49%) from KPK, 2 (4.08%) from Islamabad, 2 (4.08%) from Sindh, 2 (4.08%) from AJK/GB, and 1 (2.04%) from Balochistan.

R1939W and P1987R are biallelic variants, both having major alleles C and minor alleles T and G respectively. Allelic and genotypic frequencies in the two groups are given in Table-I. AS-PCR results of R1939W and P1987R are shown in Fig.2(a) and IIb respectively. Chi square value for the alleles of R1939W was *x²*=0.34 (*df*=1, *N*=200) with *p*=0.56, and for the alleles of P1987R was *x²*=0.02 (*df*=1, *N*=200) with *p*=0.89. In determination of association of the genotypes with NSSHL, different genetic models were adopted. These models along with ORs are given in Table-II.

**Table-I:** Allelic and genotypic frequencies of R1939W and P1987R variants of *OTOF* gene (*N*=100).

SNPs	Groups	Genotypic Frequency			Allelic Frequency	

		C/C	C/T	T/T	C	T
R1939W	Case	26% (13)	74% (37)	0	63%	37%
	Control	20% (10)	78% (39)	2% (1)	59%	41%
		C/C	C/G	G/G	C	G
P1987R	Case	10% (5)	90% (45)	0	55%	45%
	Control	12% (6)	88% (44)	0	56%	44%

**Fig.2(a):**
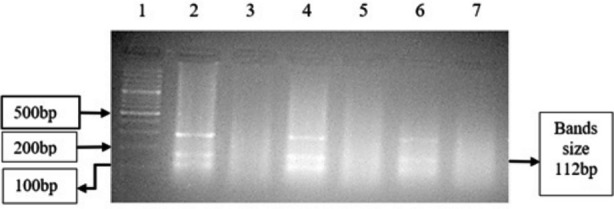
Allele-specific PCR result of R1939W variant *OTOF* gene.

**Fig.2(b):**
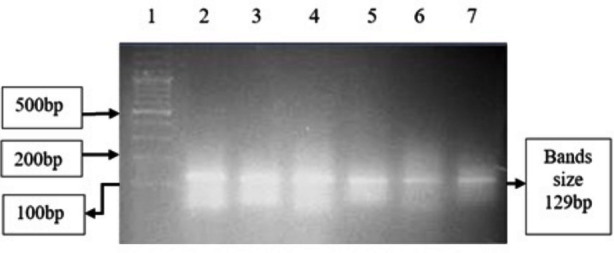
Allele-specific PCR result of P1987R variant *OTOF* gene.

**Table-II:** Genetic models used to study the association of R1939W and P1987R with NSSHL (N=100, 95% CI, adjusted by age + sex).

Model	Genotype	OR	95% CI	AIC	BIC

R1939W					
Dominant	C/C C/T-T/T	0.71	0.28-1.82	10	23
Recessive	C/C-C/T T/T	NA	NA	10	23
Overdominant	C/C-T/T C/T	0.80	0.32-2.02	10	23
P1987R					
Log-additive	C/C C/G	0.96	0.55-1.68	10	23

OR: Odds Ratio, CI: Confidence Interval, AIC: Akaike’s Information Criterion, BIC: Bayesian Information Criterion.

**Fig.3:**
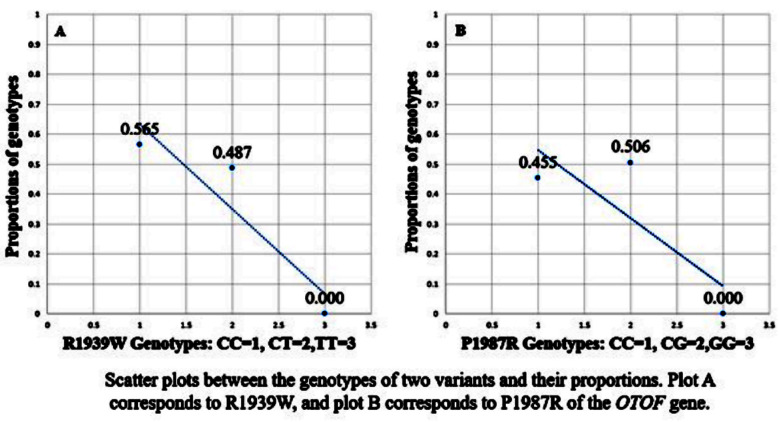
Scatter plots showing correlation between the two variants of *OTOF* gene and nonsyndromic sensorineural hearing loss.

Correlation analysis of R1939W showed *Z* (observed)=-0.91 with *p* (two-tailed) = 0.36. For P1987R, *Z* (observed) was 0.32 with *p* (two-tailed) = 0.75. Fig.3 shows scatter plots for the two variants. *P*-values for HWE were <0.0001 for both variants in the two groups. LD calculation for major alleles of the two variants showed *D’*=0.92 and *r²* (correlation coefficient) = 0.46, where haplotype frequency of CC was 0.165.

## DISCUSSION

All cases had prelingual onset of NSSHL, with increased prevalence in males (58%). Parental consanguinity was 72%, and 62% of the cases had family relatives affected with HL. Mean maternal marriage age was 22.10 years.

There was no significant association of R1939W alleles with NSSHL as given by *p* value of 0.56 i.e., > 0.05. Alleles of P1987R were also not associated with the disease (*p* =0.89). Similar results were obtained using trend test i.e., *p*=0.36 for R1939W and *p*=0.75 for P1987R, showing absence of significant association with the disease.

For the association analysis of R1939W, three best genetic models were dominant, recessive and overdominant. According to the dominant model, the genotypes C/T-T/T were negatively associated with NSSHL (OR=0.71), indicating their protective roles against the disease. OR could not be calculated for the recessive model as genotype T/T could not be detected in the case group. In the overdominant model, the heterozygote C/T also had negative association with NSSHL (OR=0.80). Log-additive was the best model for P1987R, that showed decreased risk of disease with the genotype C/G (OR=0.96). These findings suggest that NSSHL is less likely to occur in the presence of these two variants of *OTOF* gene. The scatter plots (Fig.3) show decreasing trends for both the variants, suggesting negative correlation between the genotypes and their observed proportions.

Allelic frequencies of both the variants were in Hardy-Weinberg proportion of p + q=1. *P*-value of <0.0001 indicated that the genotypic frequencies were not in Hardy-Weinberg Equilibrium of p^2^ 2pq q^2^. Linkage disequilibrium calculation showed D’=0.92, which is very close to one, indicating a high degree of correlation between major alleles of the two variants. Correlation coefficient *r²* was 0.46, meaning that the genotype of one variant could be predicted in 46/100 individuals if the other variant was known.

There seems to be an increased prevalence of hereditary deafness among CI candidates, as shown by prelingual onset of HL, significant family history and parental consanguinity. The most common inheritance pattern of NSSHL is autosomal recessive,^15^ where disease occurs when both parents carry the same recessive alleles at a locus, the probability of which increases with cousin marriages^16^, a prevalent phenomenon in our country.^6^ Also, about half of the HL cases have a genetic etiology,^17^ that usually presents early in life and tends to run in families, thus emphasizing the need for early detection, management and hence restoration of speech and language skills. Increased prevalence of HL in males is also confirmed by a US study, where males were affected twice as often as females.^18^ About 61% Pakistani females get married by the age 22 years and median age at first birth is 22.8 years,^19^ this indicates that advanced maternal age as risk factor for genetic disorders is not prevalent in our region.

The results of association analysis are contrary to a Pakistani study in which R1939W was found to be a causative factor for nonsyndromic hereditary HL.^20^ An American study found heterozygous mutation of P1987R to be associated with nonsyndromic auditory neuropathy.^21^ A high value of D’ indicates that the alleles of the two variants are inherited together in our population as a haplotype, suggesting only few recombination events between them.^22^

R1939W and P1987R variants of *OTOF* gene were found to be protective against NSSHL in our study subjects, suggesting other pathogenic variants of this gene or other genes^23^,^24^ as probable risk factors for hereditary HL in Pakistani population. This study has added to the national database of single nucleotide polymorphisms associated with HL, that will aid in selection of variants for future genetic testing and screening of hereditary HL. The genetic analysis data can be used as a reference for pharmacogenetic research and future genetic therapies. Furthermore, linkage disequilibrium data can be used in genome wide association studies (GWAS), for identification of risk alleles associated with HL in our population.

As consanguinity is the major underlying contributor to hereditary deafness, sharing results with the clinicians will help in establishing genetic counselling programmes with special focus on cousin marriages and disease risk estimation in future generations. There is a need of pre-marital screening and carrier detection of at-risk family members as is currently being done for thalassemia under Sindh Prevention and Control of Thalassemia Act, 2013.

### Limitations of the study:

1. Sample size was small as few cochlear implant centers are present to manage deafness in our country.
2. Family members of deaf children could not be sampled due to financial constraints.
3. Other *otoferlin* gene variants could not be studied due to limited research budget.

## CONCLUSION

NSSHL was more prevalent in males, presenting in early years of life, with bilateral, prelingual and nonprogressive disease course. Increased percentage of cousin marriages and family history of HL were detected in CI subjects. Alleles of R1939W and P1987R variants of *OTOF* gene were in high LD, however, they had no association with NSSHL. Genotypes of both the variants had protective roles against the disease.

### Authors’ Contribution:

**HN, ZAB** did data collection, wet lab work, statistical analysis, manuscript writing, and final approval of manuscript.

**AR, AM,** provided technical assistance, did study design, data interpretation, data review for intellectual content and final approval of the manuscript.

All authors bear responsibility and accountability for the integrity of the work.

## References

[1] Van Camp G, Smith RJH (2018). Hereditary Hearing Loss Homepage.

[2] WORLD REPORT ON HEARING (2021). Geneva (CH):World Health Organization.

[3] Cochlear Implants (2016). Bethesda (MD):National Institute on Deafness and Other Communication Disorders, National Institute of Health.

[4] Disability Statistics:DISABLED POPULATION BY NATURE OF DISABILITY AND SEX CENSUS –1998.

[5] Zeeshan F, Bari A, Dugal MN, Saeed F (2018). Hearing impairment after acute bacterial meningitis in children. Pak J Med Sci.

[6] Pellissier H (2012). Cousin marriage-70% in Pakistan-should it be prohibited?.

[7] Yasunaga S, Grati M, Chardenoux S, Smith TN, Friedman TB, Lalwani AK (2000). OTOF encodes multiple long and short isoforms:genetic evidence that the long ones underlie recessive deafness DFNB9. Am J Hum Genet.

[8] (2003). OTOF:HUMAN PROTEIN ATLAS SUMMARY.

[9] Zhang QJ, Han B, Lan L, Zong L, Shi W, Wang HY (2016). High frequency of OTOF mutations in Chinese infants with congenital auditory neuropathy spectrum disorder. Clin Genet.

[10] Fareed M, Sharma V, Singh I, Rehman SU, Singh G, Afzal M (2021). Whole-exome sequencing reveals a rare variant of OTOF gene causing congenital non-syndromic hearing loss among large Muslim families favoring consanguinity. Front Genet.

[11] Naz S (2021). Molecular genetic landscape of hereditary hearing loss in Pakistan. Hum Genet.

[12] Ramzan M, Bashir R, Salman M, Mujtaba G, Sobreira N, Witmer PD (2020). Spectrum of genetic variants in moderate to severe sporadic hearing loss in Pakistan. Sci Rep.

[13] Ahmed A, Wang M, Khan R, Shah AA, Guo H, Malik S (2021). A splice-site variant (c.3289-1G>T) in OTOF underlies profound hearing loss in Pakistani kindred. BMC Med Genomics.

[14] Sambrook J, Russell DW (2006). Purification of nucleic acids by extraction with phenol:chloroform. CSH Protoc.

[15] Shibata SB, Shearer AE, Smith RJH Cummings otolaryngology head and neck surgery.

[16] Bener A, Mohammad RR (2017). Global distribution of consanguinity and their impact on complex diseases:Genetic disorders from an endogamous population. Egypt J Med Hum Genet.

[17] Nance WE (2003). The genetics of deafness. Ment Retard Dev Disabil Res Rev.

[18] Hoffman HJ, Dobie RA, Losonczy KG, Themann CL, Flamme GA (2017). Declining prevalence of hearing loss in US adults aged 20 to 69 years. JAMA Otolaryngol Head Neck Surg.

[19] Pakistan Demographic and Health Survey 2017-2018.

[20] Choi BY, Ahmed ZM, Riazuddin S, Bhinder MA, Shahzad M, Husnain T (2009). Identities and frequencies of mutations of the otoferlin gene (OTOF) causing DFNB9 deafness in Pakistan. Clin Genet.

[21] Varga R, Kelley PM, Keats BJ, Starr A, Leal SM, Cohn E (2003). Non-syndromic recessive auditory neuropathy is the result of mutations in the otoferlin (OTOF) gene. J Med Genet.

[22] (2023). HAPLOTYPE.

[23] Sabiha B, Ali J, Yousafzai YM, Haider SA (2019). Novel deleterious mutation in MYO7A, TH and EVC2 in two Pakistani brothers with familial deafness:Novel mutation in familial deafness. Pak J Med Sci Q.

[24] Ullah S, Aslamkhan M, Rasheed A (2015). Molecular distribution of deafness loci in various ethnic groups of the Punjab, Pakistan. J Coll Physicians Surg Pak.

